# Berberine alleviates respiratory syncytial virus (RSV)-induced pediatric bronchiolitis and fibrosis via suppressing the HMGB1/TLR4/NF-κB pathway

**DOI:** 10.1128/spectrum.00900-25

**Published:** 2025-06-17

**Authors:** Yang Jiao, Rui Yang

**Affiliations:** 1 Department of Pediatrics, Affiliated Hospital of Shandong University of Traditional Chinese Medicine 680869, Jinan, Shandong, China; 2 Department of Pediatrics, Hebei Reproductive Health Hospital, Hebei Women and Children's Health Hospital, Shijiazhuang, Hebei, China; Regional Centre for Biotechnology, Faridabad, Haryana, India

**Keywords:** respiratory syncytial virus, pediatric bronchiolitis, berberine, inflammation, epithelial-mesenchymal transformation

## Abstract

Berberine exhibits antitumor, antibacterial, and antiviral properties. This study explored its therapeutic potential and molecular mechanism in respiratory syncytial virus (RSV)-induced pediatric bronchiolitis (PB). BEAS-2B cells were infected with RSV (multiplicity of infection = 0.5, 1.0, or 5.0) to establish a PB cell model. Quantitative reverse transcription PCR (qRT-PCR) quantified RSV, HMGB1, and fibrosis-related genes. MTT assay assessed cell viability, and plaque assay measured RSV titers. Western blot, immunohistochemistry (IHC), immunofluorescence, and enzyme-linked immunosorbent assay (ELISA) evaluated HMGB1 and key markers of inflammation, pyroptosis, epithelial-mesenchymal transition (EMT), and fibrosis. Flow cytometry analyzed apoptosis, while electron microscopy visualized RSV-induced pyroptotic and apoptotic changes. A PB mouse model was established by administering RSV (6.8 × 10⁶ PFU) via nasal drip in BALB/c mice. Mice were divided into six groups (*n* = 5 per group): blank, RSV, RSV + dimethyl sulfoxide (DMSO), RSV + berberine low dose, RSV + berberine medium dose and RSV + berberine high dose. HE staining examined lung pathology, while Western blot, IHC, ELISA, and immunofluorescence quantified HMGB1 and markers of inflammation, pyroptosis, EMT, and fibrosis. Berberine significantly reduced RSV replication, alleviated lung inflammation and fibrosis, and suppressed the HMGB1/TLR4/NF-κB pathway in both RSV-infected BEAS-2B cells and PB mice, as indicated by the downregulation of HMGB1, TLR4, p-IκBα, and p-p65. Berberine also inhibited pyroptosis (NLRP3, ASC, cleaved caspase-1, caspase-11, and GSDMD-N), EMT (Snail and N-cadherin), and fibrosis (CoL1A1, α-SMA, fibronectin, and ACTA2). Electron microscopy confirmed that RSV-induced cellular damage, including mitochondrial swelling and chromatin condensation, was alleviated by berberine treatment. These protective effects were reversed by HMGB1 overexpression and enhanced by TLR4 inhibition, indicating that berberine exerted its effects via the HMGB1/TLR4/NF-κB signaling pathway. Overall, berberine attenuated RSV-induced PB by suppressing the HMGB1/TLR4/NF-κB pathway, reducing inflammation, pyroptosis, EMT, and fibrosis in lung tissues and epithelial cells, which may be a promising remedy for RSV-induced PB.

**IMPORTANCE:**

Pediatric bronchiolitis caused by RSV remains a major global health challenge with limited treatment options. This study highlights berberine as a promising therapeutic agent capable of reducing RSV replication and associated lung injury. By targeting the HMGB1/TLR4/NF-κB pathway, berberine effectively attenuates inflammation, pyroptosis, EMT, and fibrosis. These findings provide a novel mechanistic insight and offer potential for the development of targeted therapies against RSV-induced bronchiolitis.

## INTRODUCTION

Pediatric bronchiolitis (PB) represents a frequently encountered respiratory illness in early childhood, marked by acute inflammatory changes in the bronchioles. Among its viral causes, respiratory syncytial virus (RSV) serves as the predominant etiological agent implicated in pediatric bronchiolitis, contributing to a substantial proportion of clinical presentations (1, 2). RSV infection activates multiple inflammatory pathways leading to excessive inflammatory cytokine release and airway epithelial damage (3). Additionally, chronic inflammation promotes epithelial-mesenchymal transition (EMT), facilitating extracellular matrix deposition and lung tissue fibrosis (4). This process ultimately contributes to pulmonary dysfunction, including atelectasis and airway obstruction (5, 6). Despite advancements in supportive care, effective targeted treatments for RSV-induced PB remain lacking (7, 8). Given the high global incidence—approximately 30 million new cases and 100,000 deaths annually (9)—new therapeutic strategies are urgently needed.

Berberine (BBR) is an alkaloid derived from medicinal herbs and has demonstrated broad pharmacological activities (10, 11). Several reports indicated its inhibitory impact on replication of herpes simplex virus, adenovirus, and rotavirus (12–14). Moreover, berberine suppresses RSV replication and reduces inflammation in airway epithelial cells (15, 16). It has also been found to ameliorate fibrosis in various conditions, such as myocardial ischemia and pulmonary fibrosis (17). However, the precise role and molecular mechanism of berberine in RSV-induced PB remain unexplored.

Recent research has identified high-mobility group box 1 (HMGB1) as a key regulator of RSV-induced lung inflammation. Studies showed increased HMGB1 levels in serum and lung tissues of PB patients (18, 19). HMGB1 activates the TLR4/NF-κB signaling pathway, which drives inflammatory responses, EMT, and fibrosis in lung epithelial cells (20, 21). Notably, berberine has been shown to inhibit the HMGB1/TLR4/NF-κB pathway in other inflammatory diseases, such as myocardial ischemia and neuroinflammation (22, 23). However, whether berberine can suppress RSV-induced PB via HMGB1/TLR4/NF-κB inhibition has not yet been investigated.

We investigated the therapeutic efficacy of berberine in RSV-induced PB and elucidated its intrinsic molecular processes. Using *in vitro* (BEAS-2B cells) and *in vivo* (RSV-infected mice) models, we investigated whether berberine could attenuate inflammation, EMT, and fibrosis by modulating the HMGB1/TLR4/NF-κB signaling pathway. These findings offer novel perspectives into the therapeutic impact of berberine on RSV-induced pediatric bronchiolitis.

## MATERIALS AND METHODS

### Cell culture and treatment

BEAS-2B cells were cultured in 10% Dulbecco's modified Eagle medium (DMEM) and were maintained in a 5% CO_₂_ incubator. To establish a pediatric bronchiolitis (PB) cell model, BEAS-2B cells were infected with respiratory syncytial virus (RSV) (American Type Culture Collection) at different multiplicities of infection (MOI = 0.5, 1.0, or 5.0) for 12, 24, 48, and 72 h (24). To assess the effects of berberine (#B3412, Sigma-Aldrich), cells were treated with 0.1, 1.0, and 10.0 µM berberine for 12, 24, 48, and 72 h (16).

### Quantitative reverse transcription PCR

TRIzol was used for RNA isolation, followed by quantification with NanoDrop 2000 (Thermo Fisher). cDNA was prepared using PrimeScript RT reagent kit (Solarbio). Quantitative reverse transcription PCR (qRT-PCR) was conducted with SYBR Premix under standard cycling conditions. Gene expression was measured using the 2^ΔΔCt^ method. The primers are listed in Table 1.

**TABLE 1:** Primers of genes used for qRT-PCR^*a*^

Gene name	Forward	Reverse
RSV	TGGGAGARGTRGCTCCAGAATACAGGC	ARCATYACTTGCCCTGMACCATAGGC
HMGB1	TTTCAAACAAAGATGCTCACA	GTTCCCTAAACTCCTAAGCAGATA
GAPDH	GGTCATCTTCAGTTCAACA	GTGAGAGTGACTCTCTTACT
CoL1A1	CAGCCGCTTCACCTACAGC	TTTTGTATTCAATCACTGTCTTGCC
α-SMA	GCTGCTGACCGAGCGTGGCT	GTAGGTGGTTTCGTGGATGC
Fibronectin	AGACAGGACACCAGGAGCTG	CTTGCTGGTAGGAGGCAAGC
ACTA2	CCCAGACATCAGGGAGTGAT	TGTTTGCTGATGGCATGGTT

^*a*^ R, A or G; Y, T or C; M, A or C.

### Cell transfection

We transfected overexpression and control plasmids to investigate HMGB1 function in RSV-induced BEAS-2B cells. The pcDNA3.1-HMGB1 (full-length HMGB1) and pcDNA3.1-negative control plasmids were used for transfection, which was performed using Lipofectamine 3000 for 48 h, after which BEAS-2B cells were collected for further analysis. Additionally, some cells were exposed to 100 nM TLR4 inhibitor (TAK-242; Takeda, Osaka, Japan) for 15 min post-berberine treatment for downstream signaling mechanism (25).

### MTT assay

Cell viability was recorded with the MTT assay in which 1 million BEAS-2B cells grown in 96-well plates were treated with MTT solution for a few hours. Formazan crystals were solubilized in 160 µL dimethyl sulfoxide (DMSO), and optical density was read at 490 nm on a plate reader (MD, Menlo Park, CA, USA).

### Plaque assay

BEAS-2B cells (12-well plates) were infected with RSV (MOI = 0.5) for 24 h. After infection, cells were washed and treated with 1 µM berberine or DMSO for 72 h. The cells were then overlaid with DMEM containing 0.9% methylcellulose and incubated for 4–5 days. Cells were then fixed and stained with 1% crystal violet, and viral plaques were enumerated microscopically.

### Flow cytometry

Apoptosis was evaluated using an Annexin V-FITC kit (BD, Mountain View, CA, USA); BEAS-2B cells were harvested, resuspended in binding buffer, and stained with Annexin V-FITC and propidium iodide for 30 min in darkness before flow cytometric analysis (Beckman Coulter, Fullerton, CA, USA).

### Western blot

Protein from cells and tissue samples was collected and quantified with bicinchoninic acid assay kit (Vigorous Biotechnology, Beijing, China). Proteins were electrophoretically separated, transferred to polyvinylidene fluoride (PVDF) membranes, blocked in milk, and probed overnight with antibodies: HMGB1 (ab79823, Abcam), TLR4 (ab13556, Abcam), NLRP3 (ab263899, Abcam), ASC (ab155970, Abcam), cleaved caspase 1 (89332, Cell Signaling Technology), caspase 11 GAM (ab180673, Abcam), E-cadherin (ECD-H5256, AcroBiosystems), N-cadherin (sc-8424, Santa Cruz), Snail (sc-393172, Santa Cruz), IκBα (MA5-15224, Thermo Fisher), p-IκBα (ZRB1665, Sigma), p65 (#514451, Santa Cruz), p-p65 (#3033, CST), and GSDMD-N (DF13758, Biosciences) were added to incubate overnight. Following Tris-buffered saline with Tween 20 (TBST) washes, membranes were treated with secondary antibodies (#31460 and 31430, Thermo Fisher) for 1 h, and immunoreactive signals were recorded with ECL reagent (Solarbio).

### Enzyme-linked immunosorbent assay

The concentrations of inflammatory cytokines (IL-1β [#88-7013A-88, Sigma], IL-18 [#BMS618-3, Sigma], IL-33 [#M3300, #D3300B, R&D Systems], and tumor necrosis factor alpha [TNF-α; #BMS607-3, Sigma]) and fibrosis markers (CoL1A1 [#EEL218, Sigma], α-SMA [#MBS701448, Biosciences], fibronectin [#EEL096, Sigma], and ACTA2 [#LS-F35375, LS Bio]) were measured in cell culture supernatants and mouse serum using enzyme-linked immunosorbent assay (ELISA) kits. After adding the stopping solution, the optical density at 450 nm was recorded with a microplate reader (MD).

### Immunofluorescent assay

Cells were adhered to the cell culture plate by fixing them using paraformaldehyde. These cells were later permeabilized and blocked with 1% goat serum. Cells were incubated overnight with anti-CoL1A1 (1:200, #72026; CST), anti-α-SMA (1:200, ab5694; Abcam). Following phosphate-buffered saline (PBS) washes, these cells were treated with secondary antibody (1:500, #A-21211; Sigma) for 1 h. Finally, nuclei of cells were stained blue with 4′,6-diamidino-2-phenylindole (DAPI), and resultant cell images were taken with a microscope.

### RSV-induced PB mice model

Female BALB/c mice (6 weeks old) were obtained from the Chinese Academy of Sciences. Mice were divided into six groups (*n* = 5 per group): blank group (no infection), RSV group (infected with RSV), RSV + DMSO/vehicle group (RSV infection + 1% DMSO in corn oil by oral gavage), RSV + BBR low-dose group (RSV infection + berberine 10 mg/kg/day), RSV + BBR medium-dose group (RSV infection + berberine 20 mg/kg/day), and RSV + BBR high-dose group (RSV infection + berberine 30 mg/kg/day). All mice (except the blank group) were anesthetized with isoflurane and inoculated intranasally with 50 µL RSV solution (6.8 × 10⁶ PFU). DMSO-treated mice received 100 µL vehicle (1% DMSO), while berberine-treated groups received respective doses via oral administration for five consecutive days following RSV infection (26). On day 5, mice were anesthetized with 3% isoflurane, and serum was collected for ELISA analysis. Mice were then sacrificed by 2.5% isoflurane and cervical dislocation.

### Immunohistochemistry and hematoxylin-eosin staining

Lung tissues were embedded in paraffin and sectioned. The sections were then deparaffinized and rehydrated. For hematoxylin-eosin staining, the lung sections were treated with hematoxylin for 1 min and washed with tap water, and the cytoplasm of the tissues was finally stained with eosin for 2 min. The sections were subsequently washed again, dried, and mounted on the slides with cover slips. After complete drying, the pathological changes were examined under a light microscope.

For immunohistochemistry (IHC) staining, peroxidase activity in tissue sections was first blocked, and then the sections were blocked with 5% goat serum. After 1 h, the tissue sections were incubated overnight with antibodies: anti-HMGB1 (1:400, ab79823; Abcam) and anti-NLRP3 (1:200, ab214185; Abcam). After washing, the sections were incubated with secondary antibody (1:500, ab6112; Abcam) for 30 min. The color reaction was developed using 3,3′-diaminobenzidine (DAB), followed by counterstaining with hematoxylin for 1 min and observation under the microscope.

### Statistical analysis

The statistical analyses presented in Results were performed using GraphPad Prism version 8.0 and are expressed as mean ± standard deviation from three independent experiments. The multiple group comparison was achieved with one-way analysis of variance, followed by Tukey’s multiple comparison test for pairwise comparisons. *P* < 0.05 was considered statistically significant.

## RESULTS

### Berberine suppressed RSV replication and RSV-induced HMGB1 expression in BEAS-2B cells

**Fig 1:**
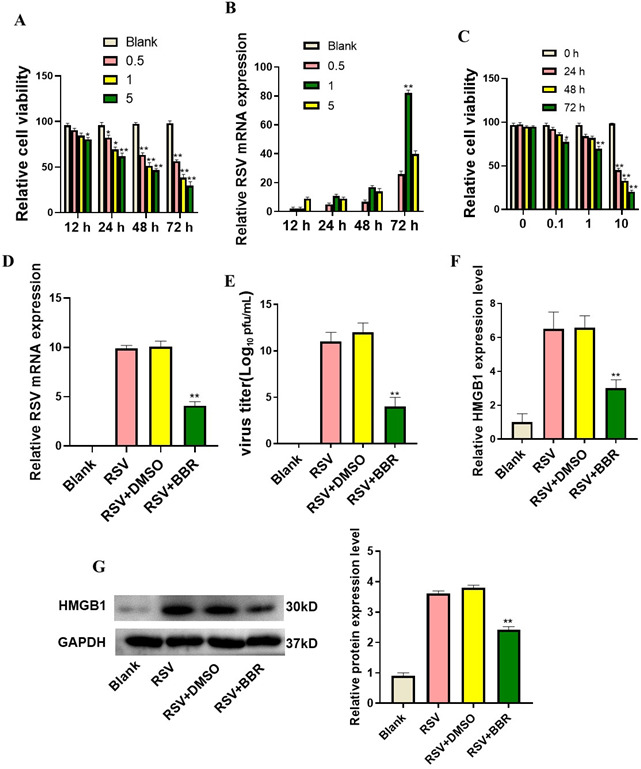
Berberine lowered RSV replication HMGB1 expression in BEAS-2B cells. (**A**) MTT assay showed BEAS-2B cell viability after RSV infection at different MOIs and time points. (**B**) qRT-PCR detected RSV levels in BEAS-2B cells post-infection. (**C**) MTT assay evaluated BEAS-2B cell viability after berberine treatment. *P < 0.05 and **P < 0.01, vs. 0 h. (**D**) qRT-PCR confirmed reduced RSV mRNA expression in BEAS-2B cells treated with berberine. (**E**) Plaque assay showed a reduction in RSV viral titers after berberine treatment. *P* < 0.01 vs. blank. (**F**) qRT-PCR analysis demonstrated decreased HMGB1 mRNA levels in RSV-infected cells after berberine treatment. (**G**) Western blot confirmed downregulation of HMGB1 protein expression in RSV-infected BEAS-2B cells treated with berberine. **P < 0.01 vs. blank.

To determine optimal MOI and infection duration for RSV in BEAS-2B cells, we first assessed cell viability and RSV mRNA expression following infection at different MOIs (0.5, 1.0, and 5.0) over 12, 24, 48, and 72 h. The results showed that RSV infection reduced cell viability, with higher MOI leading to greater viral replication (Fig. 1A). However, at MOI = 5 for 72 h, RSV replication showed a plateau, likely due to cytopathic effects (Fig. 1B). Based on these findings, we selected MOI = 1 for 72 h for subsequent experiments to balance efficient viral replication and cell viability. Next, berberine effects on cell viability and RSV replication were measured by treating BEAS-2B cells with different concentrations of berberine (0.1, 1.0, and 5.0 µM) for various durations. Cells treated with 1 µM berberine for 72 h exhibited the optimal balance between cell viability and antiviral effects and were chosen for further analysis (Fig. 1C). To confirm whether berberine inhibited RSV replication, qRT-PCR analysis was performed, which revealed a reduction in RSV mRNA levels after berberine treatment (Fig. 1D). Furthermore, a plaque assay was conducted to measure viral titers, which demonstrated a reduction in RSV particle production in berberine-treated BEAS-2B cells (Fig. 1E). In addition to RSV replication, we investigated the impact of berberine on HMGB1 expression, as HMGB1 is a key regulator of RSV-induced inflammation. Additionally, berberine treatment downregulated HMGB1 mRNA and protein expression in RSV-infected BEAS-2B cells (Fig. 1F and G). Taken together, berberine effectively suppressed the multiplication of RSV and associated HMGB1 expression.

### Berberine attenuated RSV-induced inflammation and pyroptosis of BEAS-2B cells via suppression of the HMGB1/TLR4/NF-κB pathway

**Fig 2:**
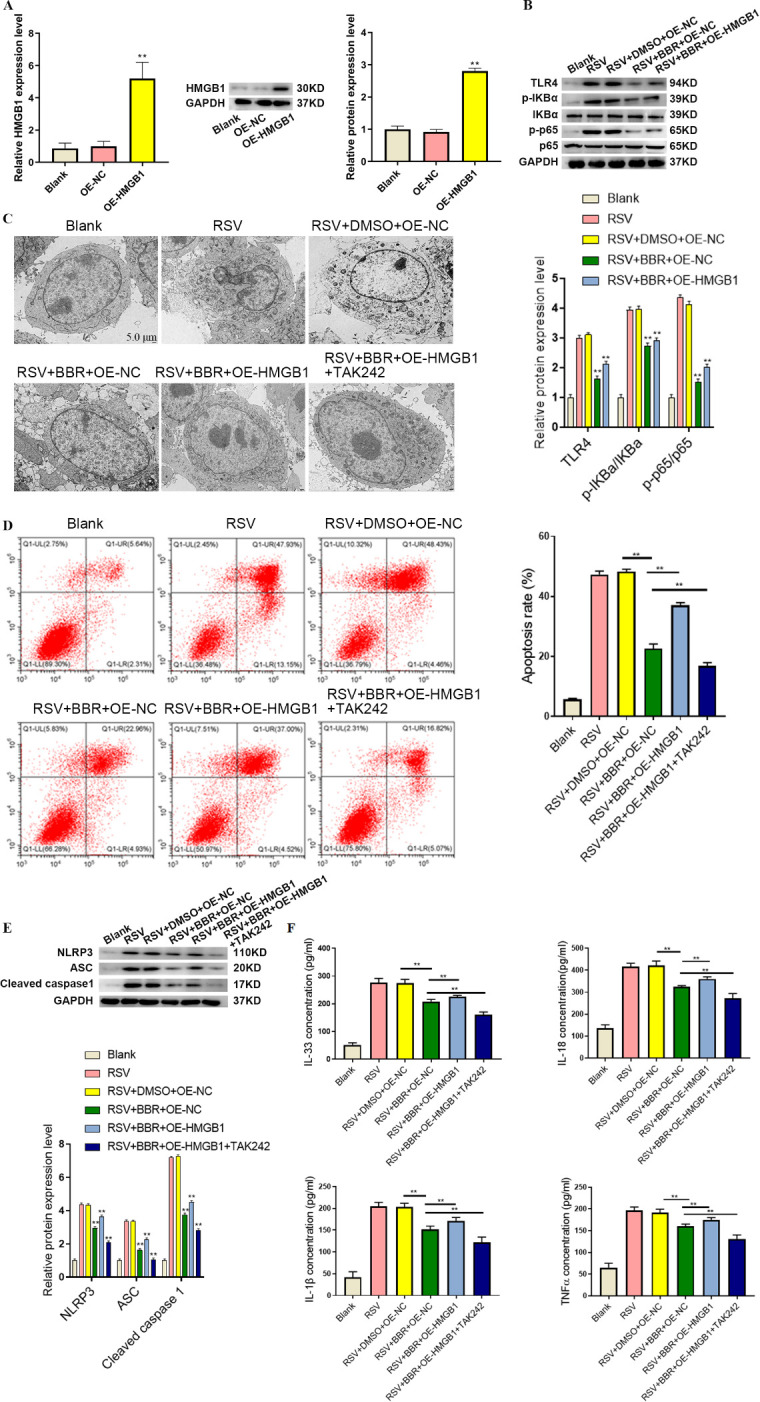
Berberine impaired RSV-induced inflammation and pyroptosis of BEAS-2B cells by inhibiting the HMGB1/TLR4/NF-kB pathway. (**A**) Relative mRNA and protein expression levels of HMGB1 in BEAS-2B cells were detected by qRT-PCR and Western blot. (**B**) The levels of TLR4, IκBα, p-IκBα, p65, and p-p65 were examined by Western blot in BEAS-2B cells. (**C**) The morphology of BEAS-2B cells was observed by an electron microscope. (**D**) The apoptosis rate of BEAS-2B cells was assessed by flow cytometry. (**E**) The protein expression of pyroptotic markers was examined by Western blot in BEAS-2B cells. (**F**) IL-1β, IL-18, IL-33, and TNF-α levels were measured by ELISA in BEAS-2B cells. **P < 0.01, vs. blank, Scale bar: 5 µm.

We next investigated the potential molecular mechanism of berberine against RSV-induced inflammation and pyroptosis in BEAS-2B cells. HMGB1 plays a crucial role in RSV-induced inflammation; therefore, we overexpressed HMGB1 (OE-HMGB1) in BEAS-2B cells. Successful HMGB1 overexpression was confirmed by utilizing both qRT-PCR and Western blots, which showed an increase in HMGB1 levels in the OE-HMGB1 group (Fig. 2A). Next, we investigated the impact of berberine on the TLR4/NF-κB signaling pathway. RSV infection led to upregulation of TLR4, p-IκBα, and p-p65, indicating pathway activation. Berberine treatment reduced these protein levels, suggesting pathway inhibition. However, HMGB1 overexpression reverted the inhibitory effects of berberine, confirming that HMGB1 mediated TLR4/NF-κB activation in RSV-infected cells. Notably, the TLR4 inhibitor TAK-242 enhanced the suppressive effects of berberine, indicating the role of TLR4 signaling in RSV-induced inflammation (Fig. 2B). We next used electron microscopy to assess cellular changes associated with RSV-induced pyroptosis (Fig. 2C). The results showed severe cellular damage in RSV-infected BEAS-2B cells, including mitochondrial swelling and nuclear disruption. Berberine treatment mitigated these structural abnormalities, while HMGB1 overexpression reversed this protective effect. Meanwhile, TLR4 inhibition further strengthened berberine’s protective effects, reinforcing the involvement of HMGB1/TLR4/NF-κB signaling in RSV-induced pyroptosis. To further validate these findings, flow cytometry analysis (Fig. 2D) demonstrated that RSV infection increased apoptosis, while berberine reduced apoptosis rates in BEAS-2B cells. However, HMGB1 overexpression reversed this effect, and TLR4 inhibition further enhanced the protective impact of berberine. Similarly, Western blots (Fig. 2E) revealed that RSV infection elevated the expression of pyroptosis markers such as NLRP3, ASC, and cleaved caspase-1, which were downregulated by berberine treatment. HMGB1 overexpression counteracted these effects and was further enhanced by TLR4 inhibition. Lastly, ELISA confirmed that RSV infection led to increased secretion of inflammatory cytokines, all of which were reduced by berberine (Fig. 2F). Again, HMGB1 overexpression reversed the suppressive effects of berberine, while TLR4 inhibition enhanced them, further validating the involvement of the HMGB1/TLR4/NF-κB pathway in RSV-induced inflammation. Overall, berberine alleviated RSV-induced inflammation and pyroptosis in BEAS-2B cells by inhibiting the HMGB1/TLR4/NF-κB pathway, with its effects reversed by HMGB1 overexpression and enhanced by TLR4 inhibition.

### Berberine reduced EMT and fibrotic factors via inhibiting the HMGB1/TLR4/NF-κB pathway in RSV-infected BEAS-2B cells

**Fig 3:**
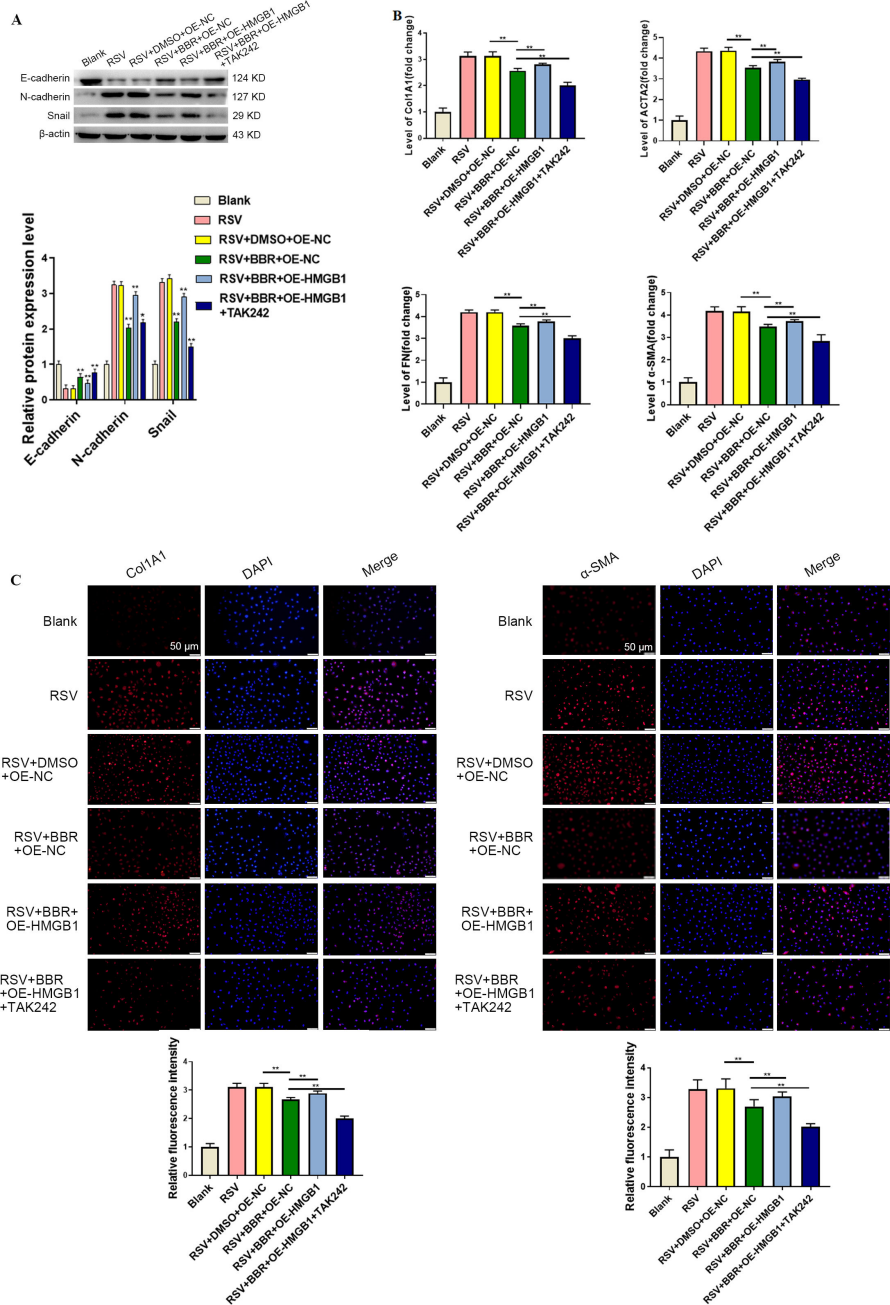
Berberine inhibited EMT and fibrosis by suppressing the HMGB1/TLR4 pathway. (**A**) Western blots of N-cadherin, Snail, and E-cadherin expression in BEAS-2B cells following RSV infection and berberine treatment. (**B**) qRT-PCR analysis of fibrotic markers in BEAS-2B cells. (**C**) Immunofluorescence analysis of CoL1A1 and α-SMA protein expression in BEAS-2B cells. *P < 0.05 and **P < 0.01. DAPI, 4′,6-diamidino-2-phenylindole.

To further investigate the effects of berberine on EMT and fibrosis, we measured EMT-associated proteins and fibrotic markers in RSV-infected BEAS-2B cells. Berberine treatment decreased N-cadherin and Snail expression but increased E-cadherin expression, indicating its inhibitory effect on EMT (Fig. 3A). Notably, these changes were partially reversed by HMGB1 overexpression and further enhanced by TLR4 inhibition. We also examined fibrosis-related markers and observed a rapid increase in CoL1A1, α-SMA, fibronectin, and ACTA2 upon RSV infection. However, berberine treatment led to downregulation of these markers, as confirmed by qPCR (Fig. 3B). Again, these effects were reversed by HMGB1 overexpression and enhanced by TLR4 inhibition. Immunofluorescence analysis further validated the berberine-induced reduction in CoL1A1 and α-SMA. HMGB1 overexpression attenuated this effect, while TLR4 inhibition restored it (Fig. 3C). Taken together, berberine mitigated EMT and fibrosis in RSV-infected BEAS-2B cells by targeting the HMGB1/TLR4/NF-κB pathway.

### Berberine suppressed the HMGB1/TLR4/NF-κB pathway to ameliorate lung injury in RSV-infected mice

**Fig 4:**
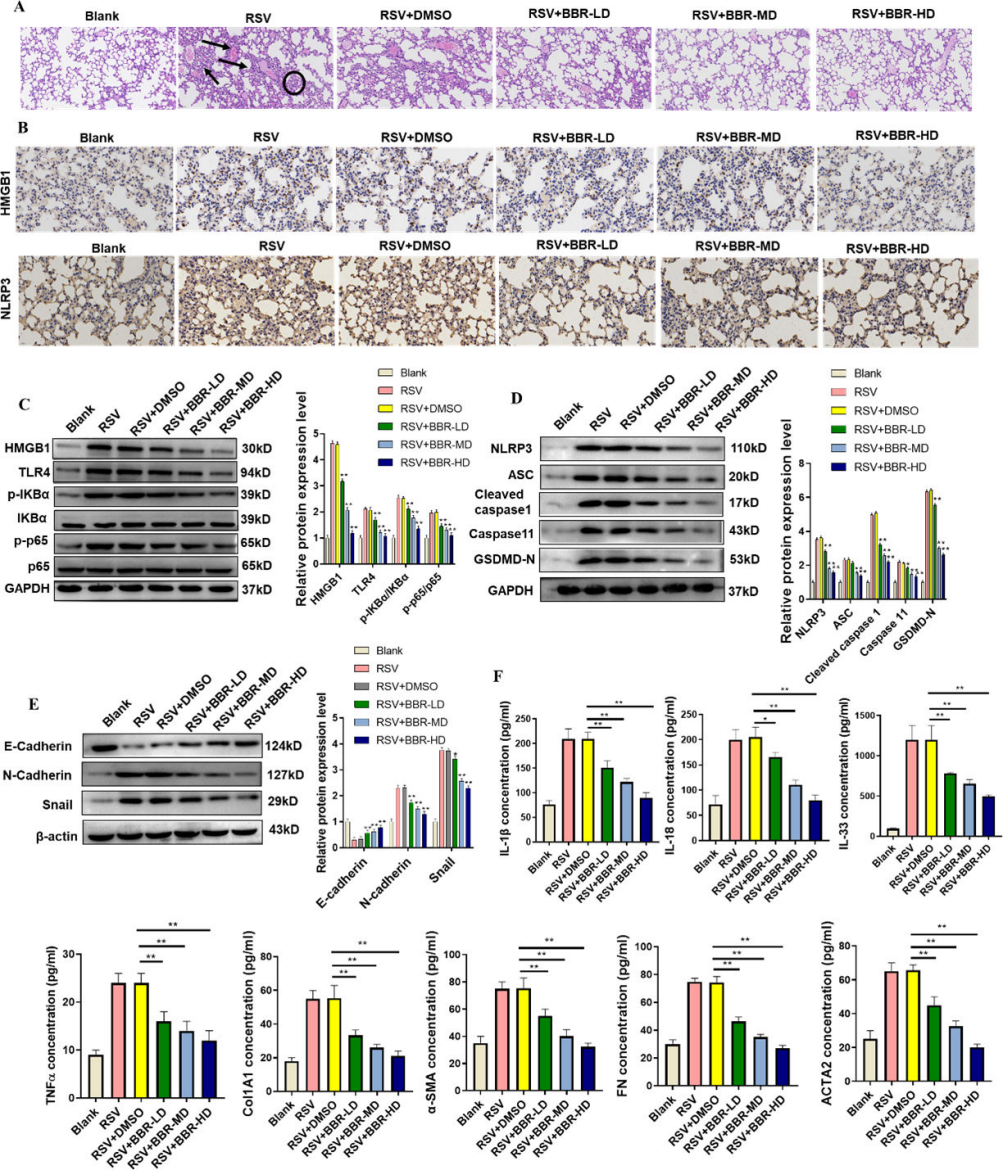
Berberine suppressed the HMGB1/TLR4/NF-κB pathway in RSV-infected mice. (**A**) Hematoxylin-eosin revealed lung tissue damage and changes in infected mice. Scale bar: 20 µm. (**B**) IHC of HMGB1 and NLRP3 in lung tissues demonstrated their increased expression following RSV infection and subsequent reduction with berberine treatment. Scale bar: 40 µm. (C–E) Western blots of NF-κB, pyroptosis, and EMT-related proteins in mouse lung tissues. (**F**) ELISA quantification of IL-1β, IL-18, IL-33, TNF-α, CoL1A1, α-SMA, fibronectin, and ACTA2 in mouse serum illustrated berberine’s dose-dependent suppression of inflammatory and fibrotic markers. *P < 0.05 and **P < 0.01. HD, high dose; LD, low dose; MD, medium dose.

To further evaluate the protective impacts of berberine, we examined its role in RSV-infected mice. RSV infection induced severe lung inflammation and injury, characterized by lymphocyte infiltration, alveolar wall thickening, and lung consolidation. However, berberine treatment mitigated these pathological changes, demonstrating its therapeutic potential (Fig. 4A). To determine whether the beneficial effects of berberine on inflammation, pyroptosis, EMT, and fibrosis were mediated via the HMGB1/TLR4/NF-κB pathway, we assessed key protein expression levels. IHC revealed that RSV infection increased HMGB1 and NLRP3 expression in lung tissues, while berberine treatment downregulated these proteins (Fig. 4B). Western blots further confirmed that RSV infection led to elevated levels of HMGB1, TLR4, phosphorylated IκBα, and phosphorylated p65, indicative of NF-κB pathway activation. However, berberine reversed these effects (Fig. 4C). We next examined the impact of berberine on pyroptosis-related markers. RSV infection increased the expression of NLRP3, ASC, cleaved caspase-1, caspase-11, and GSDMD-N, while berberine treatment effectively reversed its impacts (Fig. 4D). Regarding EMT regulation, RSV infection led to increased expression of N-cadherin and Snail, accompanied by decreased E-cadherin levels. Berberine treatment reversed these EMT-related changes, further supporting its protective effects (Fig. 4E). To assess systemic inflammation and fibrosis, we conducted ELISA analysis of serum cytokine and fibrosis marker levels. RSV infection induced upregulation of IL-1β, IL-18, IL-33, TNF-α, CoL1A1, α-SMA, fibronectin, and ACTA2, whereas berberine treatment reduced these inflammatory and fibrotic markers in a dose-dependent manner (Fig. 4F). Overall, berberine alleviated RSV-induced lung injury by suppressing the HMGB1/TLR4/NF-κB pathway, thereby inhibiting inflammation, pyroptosis, EMT, and fibrosis in RSV-infected mice.

## DISCUSSION

PB is a global public health challenge, severely affecting patient health and placing a substantial burden on healthcare systems (27). Despite considerable research efforts, effective drug therapies for RSV-induced PB remain limited (28). Previous studies have highlighted berberine’s anti-inflammatory, antineoplastic, and antiviral properties (29, 30), with reports indicating its ability to inhibit RSV replication (15). Consistently, our study confirmed that berberine reduced RSV replication in BEAS-2B cells, reinforcing its potential role in mitigating RSV-induced PB pathogenesis. Furthermore, RSV infection has been shown to induce inflammation and pyroptosis via ASC inflammasome activation and caspase-1-dependent cell death (3, 31, 32). In our study, berberine effectively attenuated RSV-induced inflammation and pyroptosis in both BEAS-2B cells and RSV-infected mice, as evidenced by reduced pyroptosis proteins and inflammatory cytokines, along with a decreased apoptosis rate. Electron microscopy further confirmed the alleviation of RSV-induced inflammation and pyroptosis following berberine treatment. These findings align with previous reports demonstrating berberine’s anti-inflammatory effects in acute lung injury and OVA-induced asthma models (33, 34).

Beyond inflammation and pyroptosis, inhibiting RSV-induced EMT and fibrosis is equally crucial for preventing disease progression (5, 6). Increasing evidence suggested that berberine alleviated fibrosis and EMT, as shown in bleomycin-induced pulmonary fibrosis, kidney disease, and adipose tissue fibrosis models (35–38). Additionally, berberine suppressed EMT by inhibiting Snail and ZEB1 expression in tumor-associated fibroblasts (39) and reversed renal tubular EMT by upregulating E-cadherin in diabetic nephropathy (40). Importantly, berberine inhibited TGF-β1-induced EMT in cancerous colon epithelial cells via inhibiting TGF-β1/Smad and NF-κB p65 pathways (41). Similarly, our study showed that berberine treatment reduced N-cadherin, Snail, and fibrosis markers while upregulating E-cadherin in RSV-infected BEAS-2B cells and mice, suggesting a protective role against RSV-induced EMT and fibrosis. Collectively, our findings indicated that berberine alleviated PB by suppressing inflammation, pyroptosis, EMT, and fibrosis.

Given the critical role of berberine, we further investigated its molecular mechanism in PB. RSV is known to trigger HMGB1 expression in PB (19), which we confirmed in this study. Importantly, berberine treatment reduced HMGB1 expression in BEAS-2B cells and mice. HMGB1 activated TLR4/NF-κB signaling, which contributed to lung injury and airway inflammation (42), and blocking the HMGB1/TLR4/NF-κB pathway mitigated lung injury and inflammation (43, 44). Additionally, berberine inhibited HMGB1/TLR4 signaling, reducing pro-inflammatory cytokines and cardiomyocyte inflammation in myocardial ischemia (22, 45). It also ameliorated cancer-derived myocardial impairment in cachexia models (46), and attenuated encephalopathy by inhibiting HMGB1/RAGE signaling (47). Berberine upregulated miR-340-5p and suppressed inflammation in myocardial ischemia and inhibited HMGB1/TLR4/NF-κB signaling in cardiomyocyte apoptosis and cancer-related myocardial impairment (45, 48). Therefore, we explored whether berberine modulated the HMGB1/TLR4/NF-κB pathway in RSV-induced PB. To further explore this, we performed *in silico* target prediction analysis using the SwissTargetPrediction, TCMSP, SEA, and PharmMapper databases, along with target gene data for RSV-induced PB from GeneCards (data not shown). HMGB1 was identified as a target of RSV-induced PB but not of berberine. This suggests that berberine likely modulates HMGB1 indirectly, potentially through upstream inhibition of RSV replication. We observed that berberine reduced concentrations of HMGB1, TLR4, p-IκBα, and p-p65, and these effects were reversed by HMGB1 overexpression in BEAS-2B cells, confirming that berberine inhibited HMGB1/TLR4/NF-κB activation in RSV-induced PB. Furthermore, we assessed whether this pathway mediates berberine’s effects on inflammation, pyroptosis, EMT, and fibrosis. Our study showed inhibitory impacts of berberine, which were reversed by HMGB1 overexpression and enhanced by TLR4 inhibition in RSV-infected BEAS-2B cells, confirming that berberine exerted its therapeutic effects via the HMGB1/TLR4/NF-κB pathway.

In conclusion, berberine alleviated pyroptosis, EMT, inflammation, and fibrosis in RSV-infected BEAS-2B cells and mice by inactivating the HMGB1/TLR4/NF-κB pathway. This demonstrated the effectiveness of berberine as a therapeutic agent for RSV-induced PB, providing valuable insights for future clinical applications.

## Data Availability

Inquiries may be directed to the authors.
